# Role of Serum Homocysteine and Outcome in Patients With Traumatic Brain Injury

**DOI:** 10.7759/cureus.28968

**Published:** 2022-09-09

**Authors:** Humayoun Amini, Hewad Hewadmal, Sayed Farhad Rasuli, Chowdhury S Shahriar, Abdul Fattah, Hariharan Kavanoor Sridhar, Marjan Khan, Sadaf Bhat, Abdul Subhan Talpur, Laila Tul Qadar

**Affiliations:** 1 Cardiology, Liaquatian Academic and Research Society, Hyderabad, PAK; 2 Cardiology, Sheikh Zayed Hospital and Medical College, Rahim Yar Khan, PAK; 3 Laparoscopic Surgery, Liaquatian Academic and Research Society, Hyderabad, PAK; 4 Internal Medicine, Liaquatian Academic and Research Society, Hyderabad, PAK; 5 Internal Medicine, Indus Medical College, Tando Muhammad Khan, PAK; 6 Anaesthesiology, Liaquatian Academic and Research Society, Hyderabad, PAK; 7 Internal Medicine, Marshfield Clinic Health System, Marshfield, USA; 8 Internal Medicine, Liaquat University of Medical and Health Sciences, Hyderabad, PAK; 9 Medicine, Liaquat University of Medical and Health Sciences, Jamshoro, PAK; 10 Internal Medicine, Dow University of Health Sciences, Karachi, PAK

**Keywords:** glasgow outcome score, traumatic brain injury, hyperhomocysteinemia, road traffic injuries, homocysteine levels

## Abstract

Background

There have been indications of a correlation between serum homocysteine (Hcy) levels and poor patient outcomes in traumatic brain injury (TBI). Thus, we aimed to explore the role of serum Hcy in influencing the outcome post TBI.

Methods

A case-control study was conducted at Liaquat University of Medical and Health Sciences (LUMHS) between January 15, 2022 and July 1, 2022. All patients between the ages of 18 and 75 years who presented with TBI, irrespective of severity, were included in the study. All patients with neurological disorders and infections, including but not limited to cerebral tuberculosis, Alzheimer's disease, epilepsy, brain cancer, Parkinson's, and stroke, were excluded from the study. For comparison, healthy controls with similar demographics were enrolled in the study. All patients and controls underwent biochemical evaluation of serum Hcy and neurological assessment at presentation. In addition, all sociodemographic and clinical parameters, including the Glasgow Outcome Score (GOS), were collected in a predefined pro forma.

Results

A total of 175 patients were included who had experienced TBIs, along with an equal number of healthy controls. The most common etiology was road traffic accidents in 82 (46.9%) patients. The mean Glasgow Coma Score (GCS) at presentation was 5.78 ± 1.72. The mean Hcy levels were 31.4 ± 7.97 µmol/L in TBI patients and 11.12 ± 5.87 µmol/L in the control healthy patients (p=0.001). It was found that the severity of hyperhomocysteinemia (HHcy) was significantly related to the worst outcome possible, i.e., death (p=0.001).

Conclusion

The study concluded that patients who had suffered from a TBI had significantly higher serum Hcy levels. Furthermore, the study highlighted that the patients with the worst outcomes had more severe hyperhomocysteinemia (HHcy) than those with better outcomes. Moreover, patients with low GOS scores were more likely to have HHcy.

## Introduction

Homocysteine (Hcy), which is an analog of the amino acid cysteine, differs because of the inclusion of a methylene bridge (-CH2-) [1]. Hcy is an essential α-amino acid formed when methionine is converted into cysteine with a total molecular mass of 135.18 g/mol. In humans, the production of serum Hcy is only possible from methionine [2]. Serum Hcy plays a major role in the human body as it contributes to many bodily functions [3,4]. Diseases like kidney dysfunction, cardiovascular disease, ischemic stroke, and neurological problems like autism can be caused as a result of high levels of Hcy in the body [5]. Several studies on rats indicate that if the levels of Hcy are elevated, it can cause seizures, Parkinson's, and related movement disorders [6]. Due to its involvement in boosting the generation of reactive oxygen species, Hcy has the potential to cause oxidative damage and damage to cell membranes [7]. Certain vitamins, such as cobalamin and pyridoxine, and also folate-rich diets can lower the Hcy levels. It is commonly found in vegetables and certain fruits [8]. Homocysteine thiolactone (HCTL), a highly reactive compound that affects the metabolic processes and physicochemical properties of peptides, is also connected with elevated Hcy levels [9]. Hyperhomocysteinemia (HHcy) is a term that refers to an anomalous elevation in Hcy levels. If total serum Hcy is elevated, it contributes to harmful effects on cells and is associated with discrete health problems [10]. One of the many causes of death and disability in adults is head injuries. Serum Hcy is utilized as a molecular marker of several neurological outcomes after head injury [1]. Dhandapani S et al. revealed that patients with traumatic brain injury (TBI) had significantly higher levels of serum Hcy (24.03 ± 16 μmol/L) than healthy individuals (16.62 ± 10.4 μmol/L). The research demonstrated that elevated levels of Hcy after brain trauma could have deleterious neurological impacts [11]. The research consisted of patients of both genders who presented within 24 hours to three months of suffering from head trauma. The authors revealed that individuals with severe trauma had high levels of serum Hcy, while those with minor injuries showed no rise in serum Hcy. The elevation in serum Hcy levels after head trauma can be because of the body's stress response, which is identical to that of subarachnoid hemorrhage (SAH). In addition, the findings revealed a correlation of serum Hcy with cognitive and neurological outcomes [11]. Similar results were found in other studies [4,6]. Despite serum Hcy becoming a key prognostic marker for imbalance in metabolism in patients with cardiovascular diseases and ischemic stroke [5,6,9], it has not been studied much in patients with TBI. As a result, the current investigation was carried out to evaluate the serum levels of Hcy upon admission in patients who had sustained a TBI.

## Materials and methods

A case-control research was undertaken at Liaquat University of Medical and Health Sciences (LUMHS) between January 15, 2022 and July 1, 2022. Before the study commenced, an ethical letter was procured from the institutional review board of LUMHS (IRB#66/2022). A non-probability convenience sampling technique was used to recruit the patients and the controls. OpenEpi online software was used to collect the minimum sample size required to test our hypothesis. Dhandapani S et al. revealed that serum Hcy levels were 36.22 ± 25.3 μmol/L in TBI patients with an unfavorable Glasgow Outcome Score (GOS) versus 22.71 ± 14.3 μmol/L in those with a better GOS [11]. Using the two-sided CI of 99% and a power of 95%, a minimum sample size of 83 for each group was obtained. All patients between the ages of 18 and 75 years who presented with TBI, irrespective of severity, were included in the study. The study excluded conditions such as cerebral malaria, Alzheimer's disease, epilepsy, brain cancer, Parkinson's, and stroke. For comparison, an equal number of healthy controls was included in the study. A case of TBI was defined as a "non-degenerative" and "non-congenital" insult to the brain from an external mechanical force, subsequently causing temporary or severe damage to cognition and neurological functions. It is generally associated with an altered state of consciousness. All patients and controls underwent biochemical evaluation of serum Hcy and neurological assessment at presentation within the first 24 hours of hospitalization using Glasgow Coma Scale (GCS). A nurse or a doctor extracted 5 ml of blood from the participants after describing the procedure to them and procuring their consent. The blood was transferred to a vial and labeled appropriately. It was then taken to the laboratory, where the levels of Hcy in the blood were determined. The severity of hyperhomocysteinemia (HHcy) was divided into three categories: moderate: 15-30 mcmol/L, intermediate: 30-100 mcmol/L, and severe: >100 mcmol/L. All sociodemographic and clinical parameters, including the GOS, were collected in a predefined pro forma. Then, at the time of hospital discharge, the prognosis was assessed using the GOS score. All data were entered into the SPSS version 23. All categorical quantities were presented as frequency and proportions, while all continuous variables were presented as mean and SD. Furthermore, a correlation between serum Hcy levels, demographics, and clinical parameters was sought using independent t-test and chi-square tests. A p-value of less than 0.05 was kept as the cut-off for statistical significance.

## Results

A total of 175 patients were included who had experienced TBI. In addition, a total of 175 healthy controls were also included. The study revealed that the mean age of the patients was 47.69 ± 10.8 years. The majority were males. The mean blood pressure of patients was lower than the controls; however, the difference was not significant. The most common etiology was road traffic accidents in 82 (46.9%) patients. The mean GCS at presentation was 5.78 ± 1.72. The mean Hcy levels were 31.4 ± 7.97 µmol/L in TBI patients and 11.12 ± 5.87 µmol/L in control healthy patients (p = 0.001), as illustrated in Table 1.

**Table 1:** Sociodemographic and clinical parameters of the participants. GCS: Glasgow Coma Scale; GOS: Glasgow Outcome Score; TBI: Traumatic brain injury.

Characteristics	TBI Patients	Control Patients	P-value
Age (years)	47.69 ± 10.8	41.94 ± 10.1	0.657
Sex			0.592
Male	96 (54.9%)	91 (52%)	
Female	79 (45.1%)	84 (48%)	
BMI (kg/m^2^)	24.81 ± 5.84	24.88 ± 5.36	0.342
Hemoglobin (g/dL)	11.23 ± 6.5	12.86 ± 9.52	0.58
Hematocrit (%)	48 ± 5	46 ± 5	0.65
C-reactive protein (mg/L)	36.5 ± 8.2	8.36 ± 5.3	<0.001
Systolic blood pressure (mm/Hg)	110.12 ± 25.43	145.44 ± 37.64	0.076
Etiology of traumatic brain injury			
Road traffic accident	82 (46.9%)	-	
Firearm injury	37 (21.1%)	-	
Fall	48 (27.4%)	-	
Other	8 (4.6%)	-	
GCS at presentation	5.78 ± 1.72	15	<0.0001
Days in hospital	12.93 ± 6.46	-	
Days in the ICU	8.28 ± 3.12	-	
Outcomes in hospitals			
Deceased	60 (34.3%)	-	
Alive	115 (65.7%)	-	
GOS at discharge			
≤ 4	85 (48.6%)	-	
> 4	90 (51.4%)	-	
Homocysteine (μmol/L)	31.4 ± 7.97	11.12 ± 5.87	0.001

Table 2 below shows the correlation between HHcy and patient outcome. It was found that the severity of HHcy was significantly related to the worst outcome possible, i.e., death (p = 0.001).

**Table 2:** Correlation between hyperhomocysteinemia and patient outcomes.

Parameters	Death	Alive	P-value
Hyperhomocysteinemia			<0.001
Moderate (15-30 μmol/L)	26 (43.3%)	83 (72.2%)	
Intermediate (30-100 μmol/L)	29 (48.4%)	29 (25.2%)	
Severe (>100 μmol/L)	5 (8.3%)	3 (2.6%)	

Table 3 illustrates the correlation between HHcy and GOS among TBI patients. It was found that most patients with GOS less than or equal to four had severe or intermediate levels of Hcy (p = 0.021).

**Table 3:** Relationship between hyperhomocysteinemia and GOS. GOS: Glasgow outcome score.

Hyperhomocysteinemia	GOS ≤ 4	GOS > 4	P-value
Moderate (15-30 μmol/L)	46 (54.2%)	63 (70%)	0.021
Intermediate (30-100 μmol/L)	32 (37.6%)	26 (28.9%)	
Severe (>100 μmol/L)	7 (8.2%)	1 (1.1%)	

## Discussion

Elevated serum Hcy is often discernibly linked with ischaemic stroke, and detecting HHcy may be an adequate way of managing stroke [12]. Serum Hcy is a sign of peroxidation that can result in the creation of lethal reactive oxygen species (ROS) and lipid peroxidation [13,14]. Elevated levels are shown to have been associated with poor patient outcomes [15]. Our study also revealed a substantial correlation between increased levels of Hcy in patients who had suffered from TBI. 

In a study by Rahmani A et al., it was found that the serum Hcy level in the TBI group was considerably greater than that in the control group (7.45 ± 13.54 mol/L, p <0.0001) (20.91 ± 15.56 mol/L, respectively) [16]. Serum Hcy levels significantly correlated with the Marshall score on CT results and the GOS score (p < 0.001 and p = 0.028, respectively). Based on the GOS score, there was also a significant distinction in the average serum Hcy among individuals who died due to TBI and individuals who survived by the end of the research period (p < 0.0001 and p = 0.054, correspondingly). The study's findings revealed a substantial correlation of serum Hcy levels with the degree of trauma and prognosis in TBI patients [16].

A study conducted by Dhandapani S et al. evaluated if there was any autonomous impact of Hcy levels on cognitive outcome in individuals who suffered a brain injury in a multivariate model [11]. Greater levels of Hcy were seen in individuals with moderate-to-severe brain injuries, acute subdural hematoma (SDH), or diffusely higher radiological grades than others. The neurological results and Hcy level were significantly correlated. When compared to patients with good GOS (mean 22.71 ± 14.3 mol/L, p = 0.03), patients with unfavorable GOS exhibited considerably higher serum Hcy levels (mean 36.22 ± 25.3 mol/L). In multiple regression, Hcy level and GOS exhibited significant associations with neurological prognosis at three months, irrespective of age, dietary habits, and imaging (p = 0.01). Following head trauma, the mean serum Hcy considerably impacts neurological prognosis and may be used as a prognostic marker [11].

Lauretta MP et al. revealed that Hcy is an independent liability for acute brain injury (ABI) and a biomarker of poor prognosis in the cases of infarction, intracerebral brain hemorrhage (ICH), SAH, and TBI [17].

Results from a study by Kwon et al. showed that the average blood homocysteine concentration was 11.4±4.7 μmol/L (mean±SD). During the first week of the study, 57 (14.4%) of the 396 participants worsened. Sixty-eight percent of early neurological deterioration occurrences happened during the first 24 hours of receiving therapy. Multivariate analysis showed that high serum Hcy levels (>10.3 mol/L) were an early marker of rapid neurological decline [18]. A study included 800 epilepsy patients to study the genetic link in epilepsy and found that the MTHFR C677T variant acts as a predisposing factor for post-traumatic epilepsy. In addition, the study found that Hcy levels are a predictor of future seizure risk post TBI [19].

In conclusion, Hcy levels affect the patient's prognosis in ABI, and in certain situations, there is a probability of recurrence, according to several studies. Serum Hcy becomes a biochemical marker that neuro-intensivists might employ as a gauge for risk classification. Furthermore, a nutraceutical approach, which consists of folic acid, vitamins B6 and B12, minimizes the possibility of thrombosis and cardiovascular and neurological dysfunction in patients with critical HHcy that were admitted for neurocritical care [20].

Like any scientific study, this study had several limitations. For instance, the study did not have a long-term follow-up. Some neurological deficit takes longer to become apparent; therefore, a study with a longer follow-up would be more beneficial.

## Conclusions

We concluded that patients who had suffered from a TBI had significantly higher serum Hcy levels. The study highlighted that the patients with the worst outcomes had more severe HHcy than those with better outcomes. Moreover, patients with low GOS scores were more likely to suffer from HHcy. Further research should be done on whether or not pre-existing levels of Hcy can be used to predict the likelihood of poor prognosis after a TBI.
